# Development of *Escherichia coli* asparaginase II for the Treatment of Acute Lymphocytic Leukemia: In Silico Reduction of asparaginase II Side Effects by a Novel Mutant (V27F)

**DOI:** 10.31557/APJCP.2021.22.4.1137

**Published:** 2021-04

**Authors:** Noeman Ardalan, Abbas Akhavan Sepahi, Ramazan Ali Khavari-Nejad

**Affiliations:** 1 *Department of Microbiology, Science and Research Branch, Islamic Azad University, Tehran, Iran. *; 2 *Department of Microbiology, Faculty of Science, North Branch, Islamic Azad University, Tehran, Iran. *; 3 *Department of Biology, Science and Research Branch, Islamic Azad University, Tehran, Iran. *

**Keywords:** Acute lymphocytic leukemia, L-asparaginase, Escherichia coli, glutaminase activity, in silico-molecular

## Abstract

Acute lymphoblastic leukemia (ALL) is a common blood disease in children that is accountable for many deaths. Due to major improvements in treatment procedures in the past 50 years, the survivability of this disease has risen dramatically to about 90 percent today. L-asparaginase (ASNase) has been used to treat ALL. The glutaminase (GLNase) activity of this enzyme causes some side effects and is unnecessary for anticancer activity. This study investigated mutagenesis in *Escherichia coli *ASNase II to find a mutant with lower GLNase activity via molecular dynamics (MD) simulation. Residues with low binding energy to asparagine (Asn) and high binding energy to glutamine (Gln) were chosen for mutagenesis. A mutant with low free binding energy to Gln was then selected for molecular docking and MD studies. The results showed that V27F is a good candidate for reducing GLNase activity and that it has little effect on enzyme ASNase activity. A simulation analysis showed that the V27F mutant was more stable than the WT ASNase and that mutagenesis was quite successful.

## Introduction

Cancer is one of the most prominent diseases in the world. It has a high mortality rate and causes about 13% more deaths than other infectious diseases (Asl et al., 2018; Fard et al., 2018; Bathula et al., 2020). Acute T-cell lymphoblastic leukemia (ALL) is a hematological cancer that results from the development of large numbers of immature lymphocytes (Bongiovanni et al., 2020; Kashef et al., 2020). ALL is the most common cancer in children and a leading cause of death in childhood, with approximately two years of treatment resulting in disease-free survival for more than 85% of patients (Sudhakar et al., 2008; Xu et al., 2020; Zekavat et al., 2020). Although the incidence of ALL in developed countries is approximately 90%, in developing countries, the incidence of this disease is especially high, and its treatment success rate is low (Li et al., 2020).

After the diagnosis of ALL, the initial treatment begins with chemotherapy. This step typically takes four weeks. Chemotherapy for ALL patients usually involves the following drugs (with or without anthracycline): corticosteroid (either prednisone or dexamethasone), vincristine, L-asparaginase (ASNase), and intrathecal chemotherapy (Rohani et al., 2017; Board, 2020). One of the main reasons for the significant improvement in the survival in ALL children is the inclusion of bacterial ASNase (with Enzyme Commission number 3.5.1.1) in treatment regimens (Hatanaka et al., 2011; Lanvers-Kaminsky et al., 2020).

It has been reported that the increased survival (>80%) of ALL patients is partly due to the use of ASNase in the drug regimen (Hunt et al., 2020). Therefore, ASNase is crucial in the treatment of ALL in children. L-asparagine (Asn) is hydrolyzed to aspartic acid and ammonia by ASNase (Baskar and George, 2016; Lanvers-Kaminsky et al., 2020). This effect of ASNase effectively depletes ALL cells that are unable to synthesize (Asn) due to enzyme deficiency. It eventually inhibits protein synthesis and stops the cell cycle (Derman et al., 2020). Unfortunately, human ASNase is not suitable for therapeutic applications because it does not show much affinity for Asn. Instead, *Escherichia coli *(*E. coli*) type II ASNase is used to treat ALL because it effectively disrupts Asn circulation and can be produced relatively easily and inexpensively (Radadiya et al., 2020).

ASNase also has glutaminase (GLNase) activity. Therefore, it also breaks down glutamine (Gln) into glutamate and ammonia. GLNase activity of ASNase is not required for anticancer activity against Asn synthetase-negative cancer cells (Freire et al., 2020). Gln is necessary for normal cell proliferation. GLNase activity leads to severe side effects associated with ASNase, which has reduced ASNase function as an antitumor drug (Prakash et al., 2020a). Elevated glutamate levels by ASNase lead to an overflow of Gln from the extravascular part into the arteries and ultimately cause severe stress (Prakash et al., 2020b). Moreover, deamination of both Asn and Gln leads to hyperammonemia which has been indicated in neurotoxicity in ALL patients (Saeed et al., 2020). Almost all side effects are mostly related to the activity of the enzyme GLNase (Reinert et al., 2006; Bunpo et al., 2008; Sanjay et al., 2017). Therefore, to overcome these side effects, reducing the GLNase activity of ASNase is absolutely necessary.

## Materials and Methods

*Structure preparation and molecular docking*

According to a paper published by Sanches et al., (2003), the PDB code for ASNase II was selected as the target enzyme to design the mutation. They used the three-dimensional structure of ASNase II with code 1NNS. 

Water molecules and non-polar hydrogen atoms were removed, and the charge of the atoms was calculated using the Gasteiger-Marsili method (Gasteiger and Marsili, 1980; Rahimi et al., 2016; Roy et al., 2016). Before the binding of the substrates, the protein structure was also optimized for molecular docking.

In other work, AutoDock Vina was used to perform the molecular docking of substrates (Asn and Gln) within the active site (Bhattacharjee and Chatterjee, 2013). Molecular docking of substrates was studied to select the best substrate state in the active site. AutoDock Vina is a free virtual molecular screening and virtual molecular presentation software. AutoDock Vina can automatically calculate the substrate-binding box (Trott and Olson, 2010). The crystalline structure of ASNase contained aspartic acid as the substrate, and, therefore, the surrounding residues were designated as the active site (Sanches et al., 2003). All default parameters were considered for molecular docking. For each substrate, the best binding mode with the lowest binding energy was selected for molecular dynamics simulations.

*Molecular dynamics (MD) simulation of wild type ASNase*

MD was performed for wild-type (WT) ASNase complexed with both Asn and Gln substrates. All MD studies were carried out using GROMACS software (version 5.1) (Van Der Spoel et al., 2005; Ardalan et al., 2018). An AMBER 99SB force field was also applied. The PROPKA 2.0 server calculated the pKa of the residues of ASNase to define ionized residues in their correct ionization state (Bas et al., 2008). The partial charge and topology files of the applied substrates were generated using ACPYPE software in the ANTECHAMBER package (Wang et al., 2001). Each system was dissolved in a cubic box of the TIP3P model water and neutralized properly using sodium and chlorine (Jorgensen et al., 1983). Structure optimization in MD was performed using the steepest descent and conjugate gradient algorithms. The computational ranges of van der Waals and electrostatic interactions were 1.4 and 0.9, respectively. PME was used to calculate electrostatic interactions (Darden et al., 1993; Mirzaie et al., 2015; Bemani et al., 2018). Meanwhile, (Mirzaie et al., 2015) utilized Berendsen for the temperature equilibrium and a Parrinello-Rahman barostat for the pressure equilibrium. A temperature of 300 K and a pressure of 1 atmosphere were considered for all MD runs, and all systems reached equilibrium at this temperature and pressure (Koushki et al., 2020). In the present study, each system was analyzed for 20 ns. The output of MD runs was analyzed using VMD software, and the diagrams were plotted using Excel.

*The free binding energy of ASNase*

MM-PBSA is a free software used to calculate the free binding energy between two defined groups. The MM-PBSA algorithm has recently been used as a scoring function in the computational drug design (Kumari et al., 2014; Mollica et al., 2016). In this study, the MM-PBSA method was used to calculate the free energy between substrates and ASNase. The free binding energy is calculated from the following formula:

Gbinding = Gcomplex - (Gprotein + Gligand)          (1)

Where Gcomplex is the total free energy of the protein-substrate complex, and Gprotein and Gligand are the total free energy of the separated protein and substrate in the solvent, respectively (Mollica et al., 2016).

*The free binding energy for each residue in the ASNase WT-substrates*

Another feature of MM-PBSA is that it calculates the free energy of binding each residue to the substrate (Ardalan et al., 2018). After calculating the energy, residues interacting more with Gln than Asn, based on their free binding energy and those not essential for the catalytic function of the enzyme, were selected for mutagenesis. Therefore, residues with a low free energy of binding to Asn and high free energy of binding to Gln were selected.

*Molecular docking, MD, and free binding energy of substrates in mutant enzymes*

The selected mutations in the enzyme crystal structure were applied by Discovery Studio software. The mutants were prepared like the WT enzyme, and the substrates were docked into the active site. MD simulations and free binding energy were performed under the same WT enzyme conditions.

*Analysis of MD simulation results of mutant and WT enzymes*

After completing MD optimization using the existing commands in GROMACS software for a series of results- including RMSD, RMSF, the intra-molecular and inter-molecular hydrogen bonds, the radius of gyration (Rg), solvent accessible surface area (SASA), principal component analysis (PCA), Dynamical cross-correlation matrix (DCCM)- the free energy landscape (FEL) obtained during the simulation was extracted for simulation analysis. Also, the residue interaction network (RIN) was created after MD simulation. The RIN of each system was designed by the NAPS webserver (Chakrabarty et al., 2019).

*Principal component analysis (PCA)*

The PCA was engaged to investigate conformational changes. As part of the PCA, a covariance matrix was constructed from the trajectories after the removal of the rotational and translational movements. The computation of the projection of eigenvalues and eigenvectors along the first two PCA was done by gmx_covar and gmx_anaeig GROMACS tools (Priya Doss et al., 2014). This procedure divided the enzymes into two conformational subspaces—the first is the essential subspace, and the second is the physically non-essential subspace (Nemaysh and Luthra, 2017). After the PCA of the backbone atoms in each system was finished, the first two eigenvectors (EigeV1 and EigeV2) were used to study FEL (Noorbakhsh et al., 2021).

*Dynamical cross-correlation matrix analysis*

Correlated motions can happen between proximal residues and also among areas as in domain-domain communication (Luo and Bruice, 2002). In the present study, the cross-correlation of the atomic fluctuations captured from the MD simulations was investigated. The dynamical cross-correlation matrix (DCCM) was computed using Equation (2) (Noorbakhsh et al., 2021):

$C_{\mathrm{ij}}=\frac{\left({∆r}_{i}.{∆r}_{i}\right)}{\left(\sqrt[{2}]{\langle {∆r}_{i}^{2}.\rangle \sqrt[{2}]{\langle {∆r}_{i}^{2}.\rangle }}\right)}$          (2)

Where i and j display *i*-th and *j*-th residues and **∆**r_i_ and **∆**r_j_ correspond to the replacement of *i*-th and *j*-th atom from the mean position, respectively (Noorbakhsh et al., 2021), the DCCM was created by an R base analysis tool (Grant et al., 2006).

## Results

*Molecular docking of WT ASNase*

The docking energy of WT with Asn and Gln substrates is 52.76 kcal mol-1 and -45.86 kcal mol-1, respectively.

*Determination of residues suitable for mutagenesis*

After performing the MD of the WT enzyme and using MM-PBSA, the binding energy of each protein residue with the enzyme substrates was investigated. Residues deemed suitable to serve as mutants in this study are listed in Table 1.

Because the main goal of this study was to reduce GLNase activity, a residue that binds more to Gln than Asn was selected. Among these residues, Gly11, Val27, and Thr166 were the most suitable residues for mutagenesis. The Tyr25, Thr12, Thr89, Asp90, and Lys162 residues have a catalytic role and bind to the substrate (Borek et al., 2014; Ardalan et al., 2018). The Ser58, Asn248, and Gln59 have a role in binding to the substrate (Sanches et al., 2003; Borek et al., 2014). Hence, they play an essential role in the catalytic activity of this enzyme, and their mutations decrease ASNase activity.

*Creating different mutations in selected residues*

The three residues were selected include Gly11, Val27, and Thr166. The selected residues were mutated into other residues whose binding energies with Asn and Gln are shown in Table 2. Based on the results, the V27F mutant was elected, which had binding energy of 13.992 Kcal mol-1 with Asn and binding energy of 3.51 Kcal mol-1 with Gln. It had lower binding energy to Gln than the WT enzyme and similar binding energy with Asn.

*Interaction of the active sites of the ASNase to Gln after MD simulation for 20 ns*

**Figure 1:**
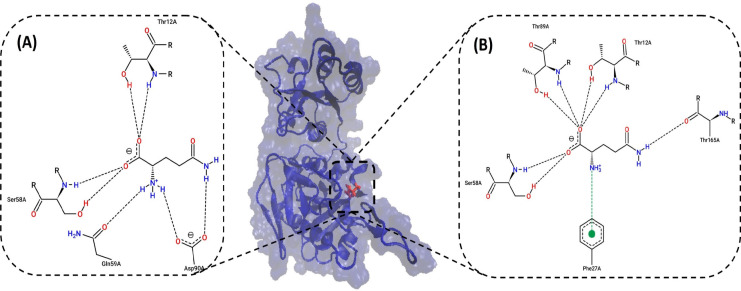
Interaction of the Gln Substrate at the Active Site of V27F ASNase (B) and WT (A).

After 20 ns MD simulation, the PDB file was taken, and the interaction of the Gln substrate in the active site of V27F was compared with that of WT (Figure 1). The decision of whether to reduce GLNase activity to reduce the ASNase activity of the mutant enzyme compared to that of the WT enzyme was based on the calculated free binding energy.

In this section, the reason for the decrease in the calculated binding energy is investigated. As shown in Figure 1, Ser58 interacts with the carboxyl group of Gln via hydrogen bonding (backbone) in the WT enzyme and V27F. Residues of the WT enzyme interact with the substrate include Thr12, Ser58, Gln59, and Asp90, all of which are essential in the ASNase active site. On the other hand, residues of V27F that interact with the substrate include Phe27, Ser58, Thr12, Thr89, and Thr165. A comparison of these residues revealed that in V27F, the residues of Gln59 (substrate-binding residue) and Asp90 (catalytic residue) no longer interacted with the substrate. Therefore, it was concluded that V27F decreases GLNase activity by reducing the interaction of two essential residues in the active site with the substrate (Gln). The numbers of hydrogen bonds in WT and mutant enzymes are 5 and 4, respectively. In V27F, a cation-π bond was established between the Phe27 and NH3 groups of Gln.

*Evaluation of the stability of the investigated systems by RMSD analysis*

**Figure 2:**
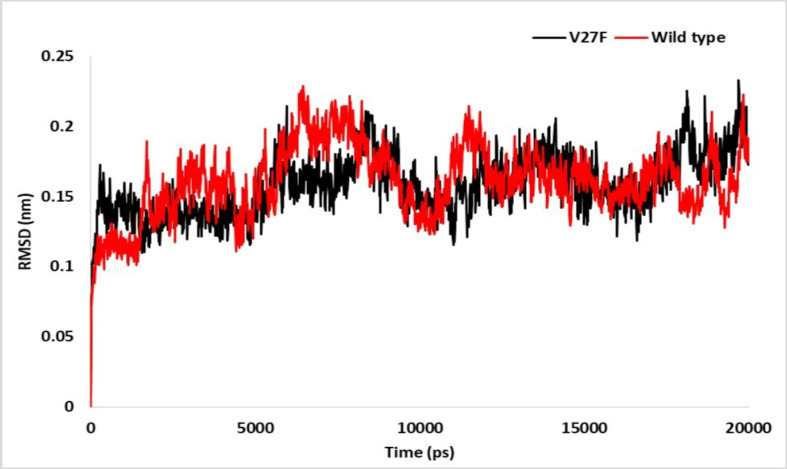
RMSD for V27F (black) and WT (red) ASNase after MD Simulation for 20 ns

Root mean square deviation (RMSD) is a standard parameter in MD studies that is measured to ensure system equilibrium during simulations. The RMSD for V27F and WT enzymes were analyzed after 20 ns of MD simulation (Figure 2). According to this figure, the RMSD values of the WT enzyme and V27F complex system in the first 1,500 ps of the simulation are different. In the WT system, the RMSD is approximately equal to 0.11 nm at the first 1,500 ps, but in the V27F system, the RMSD is about 0.14 nm at the first 1,500 ps (the value has also reached 0.16 nm at 280 ps). At 1,800 ps, the RMSD of the WT system is 0.19, which is much higher than that of the mutant system.

At up to 5,000 ps of simulation, the V27F system has a better equilibrium and stability than the WT enzyme. From 5,000-10,900 ps, the RMSD fluctuates in both systems. In the WT enzyme system, the RMSD value rose to 0.22 nm at 6460 ps and fell to 0.12 nm at 10,450 ps. Also, in the V27F system, the RMSD value increased to 0.21 nm at 5950 ps and dropped to 0.12 nm at 10,540 ps. At higher ps values, the RMSD represents a mutant system that reaches a relative equilibrium of up to 17,320 ps.

However, for the WT enzyme system, the RMSD peaked at 11,390 ps and reached relative equilibrium at 12,130 ps. Then, at 17,660 ps, the RMSD fluctuated again in both systems. The RMSD for the WT enzyme system is lower than that for the V27F system within the range of 18,000-18,720 ps. At 18,120 ps, RMSD is 0.22 and 0.14 nm in the V27F and WT systems, respectively. In general, RMSD fluctuations can be seen in both systems. Still, there are fewer abnormal fluctuations in the mutant system than in the WT enzyme. The mutant system also has a better equilibrium than the WT enzyme system.

**Table 1:** RMSD for V27F (black) and WT (red) ASNase after MD Simulation for 20 ns

Residues number	3-Letter Symbol	Energy binding to Asn (Kcal mol^-1^)	Energy binding to Gln (Kcal mol^-1^)
11	**Gly**	**0.7433**	**-3.0767**
18	Asp	-1.5354	-1.5463
27	**Val**	**0.0354**	**-0.9111**
49	Lys	-0.4175	-0.562
57	Gly	0.9027	-1.8033
79	Lys	-0.6531	-0.7074
88	Gly	-1.7244	-3.5905
166	**Thr**	**-0.2625**	**-0.7087**
167	Asp	-1.2444	-1.9781

**Figure 3:**
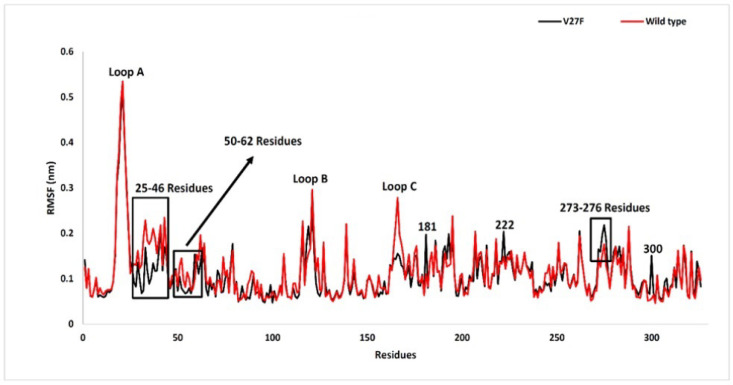
RMSF for V27F (black) and WT (red) ASNase after MD Simulation for 20 ns

**Figure 4:**
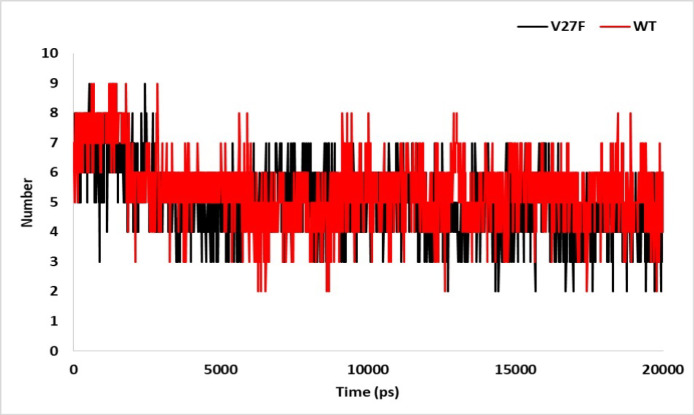
Inter-Molecular H-bond in V27F (black) and WT (red) ASNase after 20 ns of MD Simulation

**Table 2:** The Binding Energy of Mutants to Substrates Compared to WT; the Selected Mutant is Shown in Bold

Variants	Binding Energy to Asn (Kcal mol^-1^)	Binding Energy to Gln (Kcal mol^-1^)
Wild type	-15.756	-20.763
G11L	3.838	4.968
G11S	-4.354	-3.776
G11V	-12.726	-15.707
T166N	-20.902	-20.567
T166F	-14.52	-16.142
T166L	-18.669	-22.021
**V27F**	**-13.992**	**3.51**
V27L	-18.093	-3.669
V27D	-11.797	-15.735
V27K	13.911	-17.574

**Figure 5:**
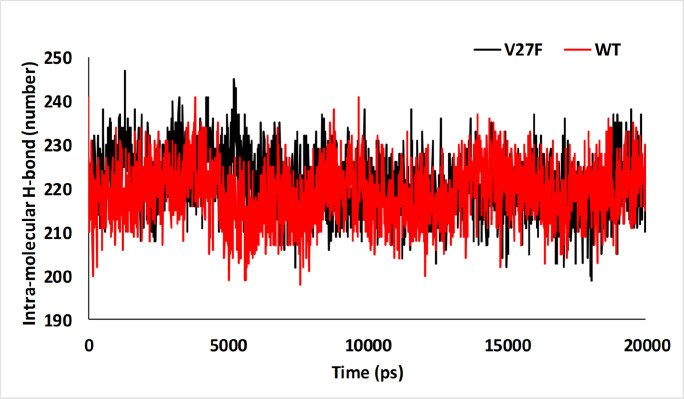
Intra-Molecular H-bond in V27F (black) and WT (red) ASNase after 20 ns of MD Simulation

**Figure 6:**
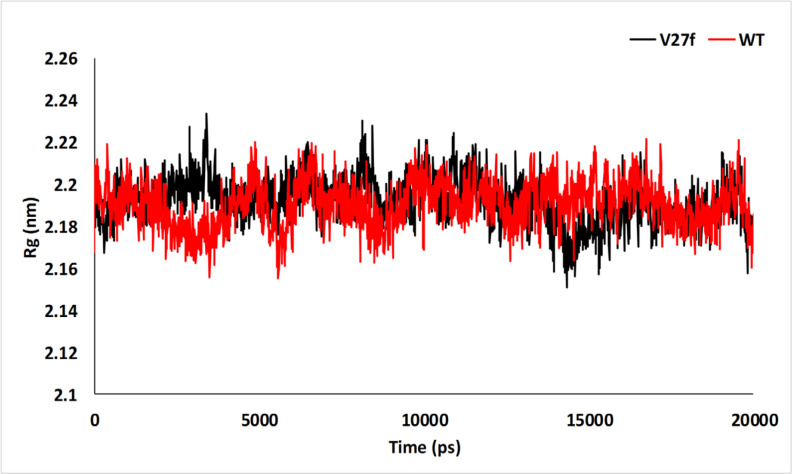
The Rg Plot for the V27F (black) and WT (red) ASNase Systems after 20 ns of MD Simulation

**Figure 7:**
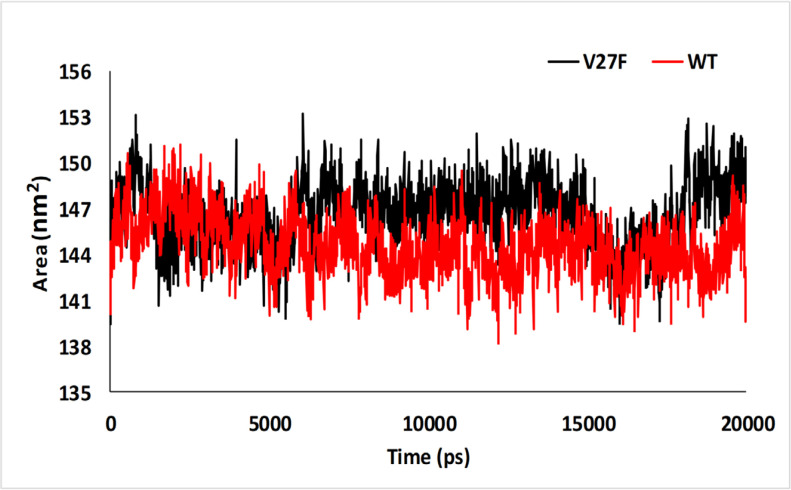
The Calculated SASA of V27F (black) and WT (red) ASNase after 20 ns of MD Simulation

**Figure 8:**
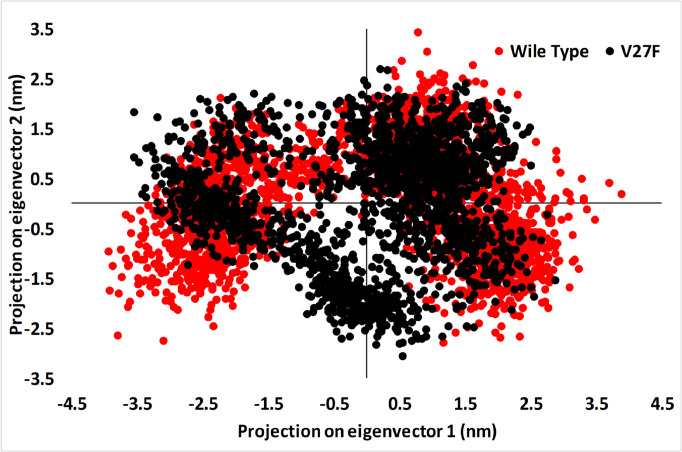
The Principal Component Analysis (PCA) of V27F (black) and WT (red) ASNase after 20 ns of MD Simulation

**Figure 9:**
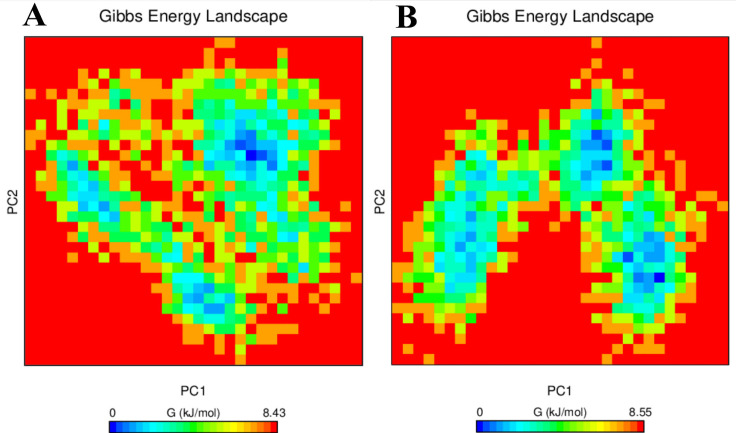
Free Energy Landscape Analysis of (A) V27F and (B) WT. The blue points demonstrate the principal components where the energy is minimum, and the red points describe the principal components where the energy is maximum.

**Figure 10:**
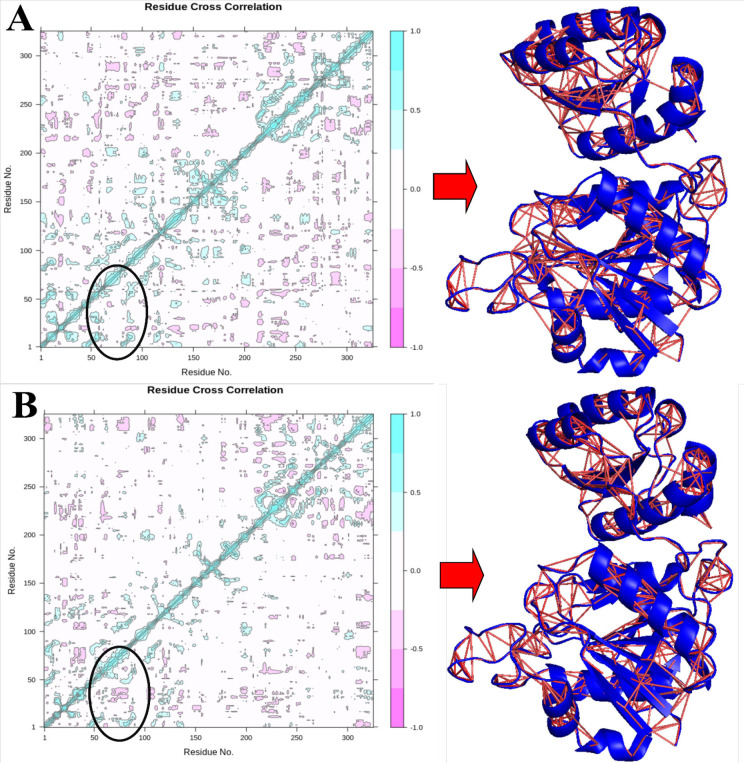
Dynamic Cross-Correlation Matrix (DCCM) of V27F (A) and WT (C), the Pink Color Indicates Negative (Anti) Correlated Motions, and the Cyan Color Shows Positively Correlated Motions. Deeper colors display a stronger correlation. Anti-correlated motions of V27F and WT are illustrated by red lines in the 3D structure of each V27F and WT ASNase protein

**Figure 11:**
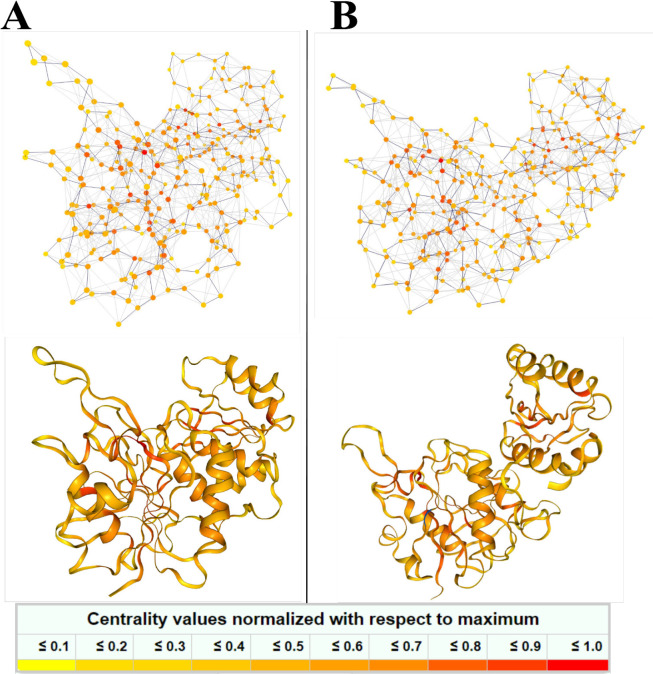
3D Residue Interaction Network (RIN) of (A) V27F and (B) WT. The nodes are colored based on their connectivity (highly connected in red color).

## Discussion

*Evaluation of the residue flexibility of the investigated systems by RMSF analysis*

Root mean square fluctuations (RMSFs) indicate the mobility and flexibility of the residues during the simulation. High RMSF values indicate high flexibility (Figure 3). It has been observed that the residue of Thr21 has the highest flexibility in both mutant and WT enzymes (5.52 Å and Å 5.34, respectively). Figure 3 shows the peak with the highest RMSF, which corresponds to three loops in the enzyme ASNase. Loop B and Loop C in the V27F have less flexibility, probably due to the mutation.

Residues 25-46 also have different levels of flexibility in the WT enzyme and V27F—the RMSFs of these residues are lower in V27F than in the WT enzyme. Mutations in the V27 enzyme also reduced the flexibility of this residue and surrounding residues.

Residues 50-62 also have different RMSFs in the two systems. Residues 58 and 59 are essential binding residues of the substrate, which is in the 50-62 range. The* V27F* mutation has reduced their flexibility. Thus, it can be said that this mutation reduces the RMSF in the two 58 and 59 residues, thereby reducing their capacity to bind to the Gln substrate. Residues with a higher RMSF in the V27F enzyme than in the WT enzyme include residues 181, 222, and 300. Residues in the range of 276-273 also have more flexibility in V27F.

*The intra- and inter-hydrogen bonds number during MD simulation*

Figure 4 shows the number of hydrogen bonds in the two investigated systems. Based on this figure, it can be observed that in some simulation times, the number of hydrogen bonds in the V27F enzyme system has decreased compared to that in the WT enzyme. Although in some other times, the number of hydrogen bonds in the V27F enzyme system has increased compared to that in the WT enzyme. In general, the average number of hydrogen bonds in the WT enzyme and the V27F enzyme systems was 5.4562 and 5,359, respectively, which indicates a relative reduction in the V27F system.

The higher number of the intramolecular H-bond indicates more stability of the protein. The intra-molecular H-bond was shown in Figure 5. The average of intra-molecular H-bond numbers in the V27F and WT systems were 221.19 and 218.99, respectively. Based on the intramolecular H-bond results shown in Figure 5, there is no significant difference between the V27F and WT; thus, they have the same stability approximately.

*The radius of gyration as an index of structure compactness*

The Rg was indicated in Figure 6 for WT and V27F systems. The Rg evident the rate of compactness in the protein structure (Farshadfar et al., 2020). The Rg values of two proteins are similar to Rg values between 21.5 and 22.3 Å. This Rg value indicated that the two studied systems achieved relatively stable conformation during the 20 ns of MD run. The average Rg values of the WT and V27F ASNase were 21.8 and 21.94 Å, respectively. The WT and V27F ASNase system represented similar Rg values. These results suggest that both systems form compact and stable complexes.

*Solvent accessible surface area analysis*

SASA analysis was extracted from MD output which is shown in Figure 7. The average SASA value was 146.58 ± 2.18 nm2 and 144.56 ± 1.99 nm2 for V27F, and WT, respectively. These values show the conformational changes occurring after the *V27F* mutation. A higher SASA value suggests the expansion of protein structure (Rahman et al., 2020). The SASA value of V27F ASNase was increased briefly as compared to the WT ASNase.

*Principal component analysis*

Overall, enzymes accomplish their specific roles through collective atomic motions. Hence, the collective atomic motion of a specific enzyme is considered as a parameter to figure out the stability of the enzyme (Amir et al., 2019). The effect of the overall motion of protein due to ligands attachment was analyzed by PCA using the construction of eigenvectors. PCA is a powerful method used for determining the rigidity of each atom and large-scale motions during MD simulation (Ndagi et al., 2020). Figure 8 displays the conformational sampling of WT and V27F ASNase in the required subspace by projecting the Cα atom along eigenvectors 1 and 2.

The results show that WT ASNase had a different conformational fluctuation in comparison with the V27F mutant (Figure 8). Both systems have good stability, but a reduction was observed in the occupied conformational space of the V27F mutant, demonstrating that the V27F mutant is more stable than WT ASNase.

*Free energy landscape analysis*

The FEL plot for PC-1 and PC-2 is displayed in Figure 9. The plot shows that the Gibbs free energy value ranges from 0 to 8.43 and 0 to 8.55 for V27F ASNase (Figure 9A) and WT ASNase (Figure 9B), respectively. Both systems displayed approximately similar Gibbs energy. The *V27F* mutation does not significantly change the global minima (blue regions) during the MD simulation, suggesting both systems are thermodynamically favorable.

*Dynamical cross-correlation matrix analysis and Residue interaction network (RIN)*

The DCCM analysis of studied ASNase systems is illustrated in Figures 10A and 10B. The cyan areas in the matrix illustrate the strongly correlated motion, while the pink areas are related to the strong anti-correlated motion of the ASNase residues. We have also shown anti-correlated motions for both systems within the 3D structure of the ASNase protein (Figure 10).

The WT and mutated ASNase displayed minor variations in the correlated and anti-correlated motions. These results elucidate that the* V27F* mutation indicated little variation in the original structure of ASNase. As can be observed, the binding region, including residues 50-100, is specified by the oval line in Figure 10. According to Figure 10, the anti-correlation of the active site of V27F ASNase is lower than that of WT. This decrease indicates a lower affinity of Gln to the V27F enzyme than to WT. Overall, considering the whole V27F structure, ASNase anti-correlation is slightly higher than WT, indicating the higher stability of V27F. However, the reduction of anti-correlation in the active site residues of V27F is very important for our study.

The residue interaction networks of both ASNases were indicated in Figure 11. The results of network analysis show that both V27F and WT ASNases have high connectivity. Therefore, it is concluded that both systems have sufficient stability.

In conclusion, ASNase (mainly derived from *E. coli*) is one of the most attractive enzymes in cancer research. In this computational study, a new mutation was proposed based on Gln free binding energy to reduce the GLNase activity of this enzyme. V27F is the best mutant in this study. V27F mutagenesis reduces Gln binding energy from -20.736 Kcal mol-1 to 3.51 Kcal mol-1. The RMSD value of the V27F mutant was lower than that of WT ASNase. The mean RMSF of the V27F enzyme (0.1091 nm) was lower than that of the WT (0.1142), indicating the higher stability of this mutation (*V27F*). In general, based on the significant similarity of the RMSF values derived from both simulation systems, mutagenesis was deemed successful. The hydrogen bonds between the enzyme and the substrate in the V27F enzyme are reduced, leading to a decrease in GLNase free binding energy. MD simulation analysis showed that the V27F mutant was more stable than the WT ASNase and that mutagenesis was successful. The ASNase-V27F enzyme is introduced in this investigation based on computer studies.

## Author Contribution Statement

All authors contributed to the study conception and design. Material preparation, data collection, and analysis were performed by Dr. Noeman Ardalan, Dr. Abbas Akhavan Sepahy, and Dr. Ramazan Ali Khavari-Nejad. The first draft of the manuscript was written by Dr. Noeman Ardalan and Dr. Abbas Akhavan Sepahy. All authors commented on previous versions of the manuscript. All authors read and approved the final manuscript.
